# Relating spatial and temporal orientation pooling to population decoding solutions in human vision

**DOI:** 10.1016/j.visres.2010.04.019

**Published:** 2010-10-28

**Authors:** Ben S. Webb, Timothy Ledgeway, Paul V. McGraw

**Affiliations:** Visual Neuroscience Group, School of Psychology, University Park, University of Nottingham, Nottingham, NG7 2RD, UK

**Keywords:** Texture, Motion, Population decoding, Summary statistics

## Abstract

Spatial pooling is often considered synonymous with averaging (or other statistical combinations) of local information contained within a complex visual image. We have recently shown, however, that spatial pooling of motion signals is better characterized in terms of optimal decoding of neuronal populations rather than image statistics (Webb et al., 2007). Here we ask which computations guide the spatial and temporal pooling of local orientation signals in human vision. The observers’ task was to discriminate which of two texture patterns had a more clockwise global orientation. *Standard* textures had a common orientation; *comparison* textures were chosen independently from a skewed (asymmetrical) probability distribution with distinct spatial or temporal statistics. We simulated observers’ performance using different estimators (vector average, winner-takes-all and maximum likelihood) to decode the orientation-tuned activity of a population of model neurons. Our results revealed that the perceived global orientation of texture patterns coincided with the mean (or vector average read-out) of orientation signals accumulated over both space and time. To reconcile these results with our previous work on direction pooling, we varied stimulus duration. Perceived global orientation was accurately predicted by a vector average read-out of orientation signals at relatively short stimulus durations and maximum likelihood read-out at longer durations. Moreover, decreasing the luminance contrast of texture patterns increased the duration of the transition from a vector average to maximum likelihood read-out. Our results suggest that direction and orientation pooling use similar probabilistic read-out strategies when sufficient time is available.

## 1 Introduction

The cortex accumulates sensory evidence from early visual areas in order to form purposeful decisions and initiate motor commands. To overcome the ambiguity inherent within early, noisy neural representations, cortical pathways combine (‘pool’) incoming visual signals. The visual system has to strike a delicate balance between combining signals from a common surface and segregating signals from the other surfaces and objects. Yet there still remains little consensus on the precise nature of the computations which govern how local visual signals are pooled across space and time.

One simple solution with substantial empirical support is that the visual system averages incoming signals in order to guide perception. (Ariely, 2001; Chong & Treisman, 2003; Cohen, Singh, & Maloney, 2008; Dakin, 1999; Dakin & Watt, 1997; Morgan, Chubb, & Solomon, 2008; Morgan, Ward, & Castet, 1998; Motoyoshi, Nishida, Sharan, & Adelson, 2007; Parkes, Lund, Angelucci, Solomon, & Morgan, 2001; Pavlovskaya, Vol, & Blum, 1992; Sharan, Li, Motoyoshi, Nishida, & Adelson, 2008; Watamaniuk & Duchon, 1992; Watt, Morgan, & Ward, 1983; Whitaker, McGraw, Pacey, & Barrett, 1996; Williams & Sekuler, 1984; Wilson, Ferrera, & Yo, 1992; Zohary, Scase, & Braddick, 1996). For example, perception of a moving surface, such as a field of dots where local motion is pooled across space or a plaid pattern where the components are pooled across orientation, frequently coincides with the vector average direction or velocity of the local samples (Kim & Wilson, 1993; Mingolla, Todd, & Norman, 1992; Watamaniuk & Duchon, 1992; Williams & Sekuler, 1984; Wilson & Kim, 1994; Wilson et al., 1992; Zohary et al., 1996). Following earlier pioneering work on reaching direction (Georgopoulos, Kettner, & Schwartz, 1988; Georgopoulos, Schwartz, & Kettner, 1986), many physiological studies have demonstrated that neurons in the motion pathway compute a vector average of velocity signals in order to guide ocular following and smooth pursuit eye movements. (Ferrera, 2000; Groh, Born, & Newsome, 1997; Huang & Lisberger, 2009; Lisberger & Ferrera, 1997; Masson, 2004; Recanzone & Wurtz, 1999; Wallace, Stone, & Masson, 2005; Yang & Lisberger, 2009). In the spatial domain, texture perception also frequently coincides with spatial summary statistics (Balas, 2006; Balas, Nakano, & Rosenholtz, 2009; Beck, 1983; Dakin, 1999; Dakin & Watt, 1997; Julesz, 1981; Keeble, Kingdom, Moulden, & Morgan, 1995; Kingdom, Hayes, & Field, 2001; Morgan et al., 1998, 2008; Parkes et al., 2001; Voorhees & Poggio, 1988). Analogous statistical processes have been invoked to explain other visual decisions, including the precision with which humans judge relative position (Watt et al., 1983; Whitaker et al., 1996), tilt (Morgan et al., 1998; Parkes et al., 2001), size (Ariely, 2001; Chong & Treisman, 2003) and surface reflectance (Motoyoshi et al., 2007; Sharan et al., 2008).

Although appealing because of it simplicity (Salinas & Abbott, 1994), linear operations like vector averaging can be biased estimators if the underlying detectors are irregularly spaced or narrowly tuned (Seung & Sompolinsky, 1993; Snippe, 1996) or the local samples are distributed asymmetrically (Webb, Ledgeway, & McGraw, 2007). For local orientation pooling with some types of moving plaid stimuli, the computations may be better characterized by the intersection of constraints (IOC) rule (Adelson & Movshon, 1982; Albright, 1984; Fennema & Thompson, 1979; Movshon, Adelson, Gizzi, & Newsome, 1986; Weiss, Simoncelli, & Adelson, 2002). The IOC is an accurate mathematical description of rigid motion, but is limited by its inability to explain non-rigid forms of motion.

An alternative to averaging and IOC is a “winner-takes-all” (WTA) or “max” rule which selects the preferred stimulus of a neuron or detector with the strongest response. This form of non-linear pooling has been successfully applied to many domains, including both spatial and motion processing (Anstis, 2009; Baldassi & Burr, 2004; Baldassi, Megna, & Burr, 2006; Baldassi & Verghese, 2002; Gheri & Baldassi, 2008; Palmer, 1994; Palmer, Ames, & Lindsey, 1993; Palmer, Verghese, & Pavel, 2000; Salzman & Newsome, 1994; Shaw, 1980, 1982; Shiu & Pashler, 1995; Solomon, Lavie, & Morgan, 1997; Verghese & Stone, 1995; Webb et al., 2007). Unlike averaging, the fidelity of a WTA estimate is much less susceptible to changes in the spacing and number of detectors (Shamir, 2006). Moreover, it is simple to implement (Baldassi & Verghese, 2002) and often as least as accurate at predicting psychophysical behavior as so called “optimal decoders”, though the estimates themselves tend to be more variable (Webb et al., 2007).

A theoretical limitation with all of the above decoding solutions is that they collapse the distributed activity of a population of neurons down to a single value to represent the “best estimate” of a stimulus. Extracting a singular estimate may not be optimal, or even desirable, under all circumstances. For example, representing multi valued stimuli, such as certain forms of transparent motion, where at least two directions can be detected at any one time (Andersen, 1989; Edwards & Greenwood, 2005) may require a more principled decoding strategy (Treue, Hol, & Rauber, 2000).

A more parsimonious formulation might be to frame pooling as a statistical inference problem (Beck et al., 2008; Deneve, Latham, & Pouget, 1999; Foldiak, 1993; Gold & Shadlen, 2001; Jazayeri & Movshon, 2006; Knill & Pouget, 2004; Ma, Beck, Latham, & Pouget, 2006; Paradiso, 1988; Pouget, Dayan, & Zemel, 2000, 2003; Pouget, Zhang, Deneve, & Latham, 1998; Sanger, 1996; Seung & Sompolinsky, 1993; Weiss & Fleet, 2002; Zemel, Dayan, & Pouget, 1998), since this allows the cortex to compute and infer the probability that a wide range of stimuli are consistent with a neural response. Optimal decoding of the distributed activity across a population of neurons can then be computed as a likelihood function, which represents the probability that each of a range of stimuli gave rise to the neural response. With access to the full likelihood function, population decoders are efficient, unbiased estimators of performance on a wide range of perceptual tasks (Deneve et al., 1999; Foldiak, 1993; Paradiso, 1988; Sanger, 1996; Seung & Sompolinsky, 1993; Weiss & Fleet, 2002). The maximum likelihood decoder, for example, accurately predicts orientation discrimination (Regan & Beverley, 1985), perceived direction (Webb et al., 2007), perceived velocity (Weiss et al., 2002) and cue combination both within (Jacobs, 1999; Landy, Maloney, Johnston, & Young, 1995) and across modalities (Alais & Burr, 2004; Ernst & Banks, 2002).

**Fig. 1:**
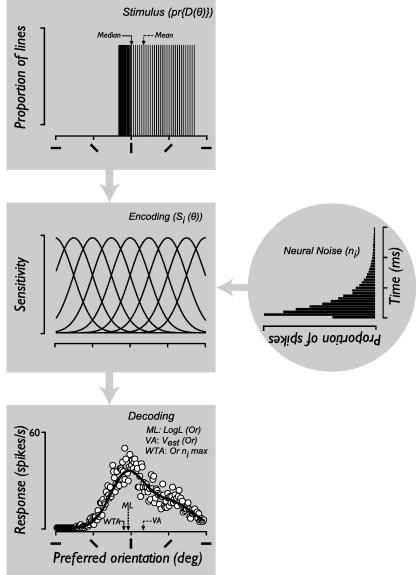
Simulation of global orientation discrimination. We simulated trial-by-trial performance on a global orientation discrimination task. A bank of orientation tuned neurons responds to an asymmetrical distribution of orientations with a Gaussian sensitivity profile corrupted by Poisson noise. From the population response, we derive the maximum likelihood, winner-take-all and vector average read-out of orientation signals accumulated over space and time.

We have developed a psychophysical paradigm that uses asymmetrical distributions of local visual signals to distinguish the contribution of different putative computations. Adopting this approach, we recently demonstrated (Webb et al., 2007) that spatial pooling of motion signals is poorly estimated by a vector average decoder, but accurately predicted by a maximum likelihood read-out of direction signals combined over space (see Fig. 1). Here we extend this paradigm to ask which class of algorithms guides the spatial and temporal pooling of local orientation signals in human vision. Our results suggest that orientation pooling uses different decoding strategies at different time scales.

## 2 Methods

### 2.1 Observers

Four observers with normal vision participated. Three were authors (BSW, PVM, TL) and one (HL) was naïve to the purpose of the experiments.

### 2.2 Stimuli

**Fig. 2:**
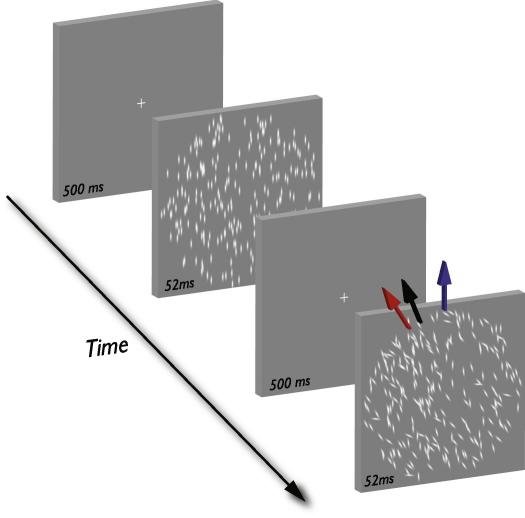
Global orientation discrimination task. The observers’ task was to discriminate which of two sequentially presented texture patterns had a more clockwise global orientation. The standard texture had a common orientation, randomly chosen from a range spanning 180°; orientations in the comparison texture were chosen independently from a skewed probability distribution with distinct spatial or temporal statistics. The mean, median and modal orientations in the comparison texture are represented by the red, black and blue arrows, respectively. This comparison texture had a clockwise SD of 30° and corresponds to the PSE data on the far right in Fig. 3A.

Static and dynamic texture patterns were generated on a *PC* computer using software written in Python using components of Psychopy (Peirce, 2007). We displayed the texture patterns on a CRT monitor (*LaCie Electron 22 Blue II or Iiyama Pro Vision Master 514*) at a viewing distance of 76.3 cm, resolution of 1280 × 1024 pixels and update rate of 75 Hz. Each texture pattern (see Fig. 2) was composed of 500 Gaussian lines (peak luminance ∼80 cd/m^2^, line envelope SD was 0.166 × 0.083°) presented on a uniform background (luminance ∼40 cd/m^2^) within a circular window (diameter 12°). *Static textures* consisted of one image; *dynamic textures* consisted of 25 images displayed consecutively at 18.75 Hz (0.052 s image duration). On each image of a dynamic sequence, lines were randomly positioned inside the circular window at non-overlapping locations.

### 2.3 Procedure

We used static and dynamic texture patterns with distinct spatial and temporal statistics, respectively. Except where stated, the procedures with both forms of texture were the same. In a temporal two-alternative forced choice task, observers judged which of two textures had a more clockwise global orientation. On each trial, we presented a standard and comparison texture in a random temporal order. Static and dynamic textures were presented for 0.052 s (1 image) and 1.3 s (25 images), respectively and separated by 0.5 s interval containing a fixation cross on a uniform background. Lines in the standard texture had a common orientation, randomly assigned on each trial from a range spanning 180°. Line orientations in the comparison texture were chosen, with replacement, from either a symmetrical or asymmetrical (skewed) probability distribution with distinct measures of central tendency. In the static and dynamic patterns, orientations were sampled from the probability distributions over space and time, respectively. A schematic of the task is shown in Fig. 2.

To investigate spatial and temporal pooling of orientation, we conducted each of the following experiments with static and dynamic textures patterns, respectively. In the first experiment, line orientations of the comparison texture were discretely sampled at 2.5° intervals from a Gaussian distribution spanning a total range of 90°. We assigned each half of the Gaussian (i.e. orientations clockwise and counter clockwise to the modal direction) a different standard deviation, thereby generating asymmetrically distributed line orientations. The standard deviation of the counter clockwise half of the Gaussian was 15°, 20°, 25° or 30°; the corresponding values for the clockwise half were 15°, 10°, 5°, or 0°.

In the second experiment, line orientations of the comparison texture were discretely sampled from a Gaussian with standard deviations of 15°, 25°, 35° or 45° for the clockwise and 6°, 10°, 14°, 18° for the counter clockwise halves. We sampled the counter clockwise and clockwise halves of the distribution at 2.5° and 0.5° intervals, respectively. This generated asymmetrical distributions of line orientations with the same mode and median but a different mean. For both experiments, the difference between the orientation of the standard texture and modal orientation of the comparison texture was varied according to the method of constant stimuli with nine levels.

In the third experiment, for the comparison texture we generated a uniform distribution of line orientations with a total range of 90°. We assigned each half of the distribution (i.e. orientations clockwise and counter clockwise to the median direction) a different range and sampling density. Line orientations for the counter clockwise half of the distribution were sampled at 2.5° intervals over a range of 45°, 55°, 65° or 75°; the corresponding values for the clockwise half were sampled over a range of 45°, 35°, 25° or 15°. This generated asymmetrical distributions of orientation with a different mean and median. The median orientation of the comparison was randomly chosen on each trial using the method of constant stimuli.

In the final two experiments, observers judged whether a static texture pattern composed of a distribution of orientations (shown in Fig. 6A; chosen from pilot work as diagnostic for distinguishing vector average and maximum likelihood read-out of orientation) was oriented clockwise or counter clockwise of implicit vertical. Each pattern was presented at seven durations, ranging between 0.05 and 3.33 s in logarithmic steps, and at three Michelson contrasts (0.25, 0.5, and 1). Global orientation was controlled via a method of constant stimuli.

### 2.4 Data analysis

For each condition, observers completed a minimum of 4 runs of 180 trials. Data were expressed as the percentage of trials on which observers judged the modal (exp. 1 and 2) or median (exp. 3–5) orientation of the comparison as more clockwise than the standard as a function of the angular difference between them and fitted with a logistic function:

(1) $$y=100/1+e^{\left(x-\mu \right)}/\theta \text{,}$$

where *y* is the percentage of clockwise judgements, *μ* is the stimulus level at which observers perceived the orientation of the standard and comparison to be the same, and *θ* is an estimate of discrimination threshold.

### 2.5 Simulations

We simulated observers’ performance on a trial-by-trial basis using the same stimulus parameters and methods described in the psychophysical procedure. The spacing and bandwidth of neurons in our model were chosen to give sufficient coverage of the orientation space. The model (shown in Fig. 1) consists of a bank of evenly spaced orientation tuned neurons spanning a 180° range. Each neuron responds to a limited range of orientations with a Gaussian sensitivity profile corrupted by Poisson noise. The separation between adjacent neurons was fixed at 1°. The sensitivity of the *i*th neuron, centered at *θi*, to orientation *θ* is:

(2) $$S_{i}(\theta )=\exp\{-[(\theta -\theta_{i})/h]{\;}^{2}\log2\}$$

where *h* is the bandwidth (half-height, half-width), fixed at 22.5° (David, Hayden, & Gallant, 2006). The response of the *i*th mechanism to stimulus *Or* with a distribution of orientations *Or*(*θ*) is:

(3) $$R_{i}(\mathrm{Or})=k\overset{180}{\underset{\theta =1}{\sum }}S_{i}(\theta )\text{pr}\{\mathrm{Or}(\theta )\}\;\text{where}\;k=R_{\max}t$$

*R_max_* is the maximum mean firing rate of the neuron in spikes/s (60), *t* is stimulus duration and pr{Or(θ)} is the proportion of orientations in the stimulus. The number of spikes (*n_i_*) elicited in response to a stimulus on a given presentation is Poisson distributed with a mean of *Ri*(*Or*)

(4) $$p(n_{i}\left|\mathrm{Or}\right))=\frac{R_{i}(\mathrm{Or}){\;}^{n_{i}}}{n_{i}!}\exp\{-R_{i}(\mathrm{Or})\}$$

The log likelihood of any stimulus *Or* is computed as a weighted sum of the responses of the population of neurons, where the activity of each neuron is multiplied by the log of its tuning function (Jazayeri & Movshon, 2006; Seung & Sompolinsky, 1993):

(5) $$\log L(\mathrm{Or})=\overset{180}{\underset{i=1}{\sum }}n_{i}\log R_{i}(\mathrm{Or})$$

The estimated orientation is the value of *θ_i_* for which logL(Or) computed for all *Or* is maximal. To obtain the estimated *Or* of the comparison from a winner-take-all decoder, we read-off the value of *θ_i_* where $n_{i}\max$. To obtain the corresponding estimate from a vector average decoder we calculated the average of the preferred orientation of all neurons weighted in proportion to their response magnitude:

(6) $$\overset{\to }{V_{\mathrm{est}}}(\mathrm{Or})=\tan^{-1}\left(\frac{\overset{180}{\underset{i=1}{\sum }}n_{i}\sin(\theta_{i})}{\overset{180}{\underset{i=1}{\sum }}n_{i}\cos(\theta_{i})}\right)$$

## 3 Results

**Fig. 3:**
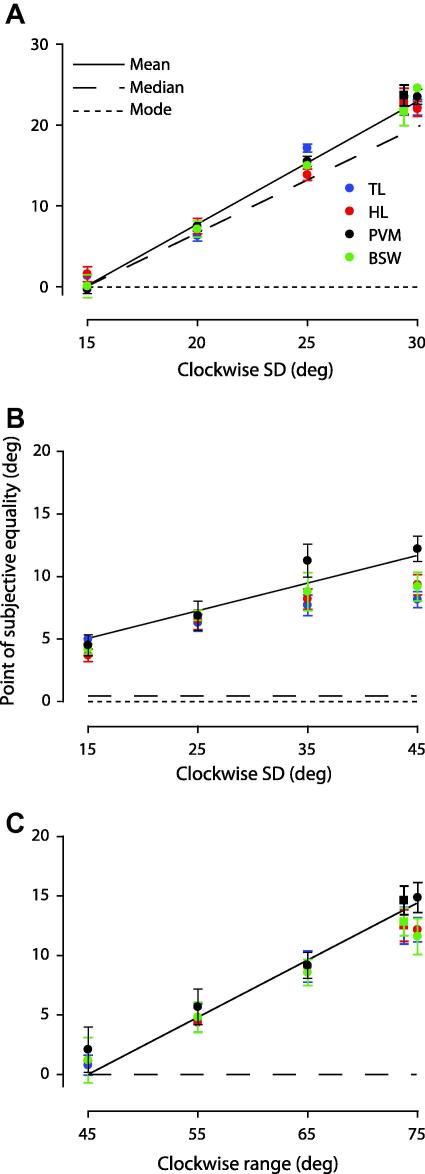
Relationship between perceived global orientation and *spatial* orientation statistics. Symbols show the point of subjective equality for four observers plotted as a function of the clockwise standard deviation (A and B) or range (C) of the comparison texture. The solid, dashed and dotted lines are respectively the mean, median and modal orientation of the comparison texture calculated across space. Error bars: 95% CIs based on 5000 bootstraps.

In the first set of experiments we investigated the pooling of orientation signals across space. We generated comparison texture patterns composed of asymmetrical distributions of orientations across space with distinct measures of central tendency. To quantify the relationship between perceived global orientation and different statistical measures of central tendency in the comparison stimulus, we estimated the point of subjective equality – the stimulus level at which observers perceived the global orientation of the comparison and standard texture to be the same (see Section 2). These data are plotted in Fig. 3 as a function of the clockwise standard deviation (A and B) or range (C) of the comparison. The perceived global orientation corresponded very closely to the mean orientation of the lines in the comparison texture. When the orientations in the comparison were drawn from a Gaussian with a clockwise standard deviation (SD) of 30° and a counter clockwise SD of 0° (comparison texture shown in Fig. 2), the modal direction of the comparison (represented by the dotted line in Fig. 3A) had to be rotated, on average, by approximately 20° to be indistinguishable from the standard (Fig. 3A). Similarly, when the comparison orientation distribution had a clockwise SD of 45° and a counter clockwise SD of 18°, the modal orientation of the comparison had to be rotated by approximately 10° to be indistinguishable from the standard (Fig. 3B). Similar effects were obtained with a skewed uniform distribution of orientations in the comparison. When the clockwise ranges were 75° and 15°, respectively, the median orientation (represented by the dashed line in Fig. 3C) had to be rotated by 13°, on average, to be indistinguishable from the standard. It is noteworthy that in Fig. 3B and C three subjects deviate slightly from the mean orientation of the comparison for the largest clockwise SD and range, respectively. The most likely reason is that some subjects reported small amounts of transparency (i.e. break down of the global orientation structure) at the largest SD and ranges.

We ran two control conditions to establish whether a vector average read-out holds when we added some orientation uncertainty to the standard stimulus. In two key conditions (comparison: counter clockwise SD 30° and clockwise SD 0°; comparison: counter clockwise range 75° and CW range 15°) the standard orientations were sampled from either a symmetrical Gaussian with a standard deviation of 30° or a uniform distribution with a range of 90°. The results for four observers are plotted in Fig. 3A and C (squares on far right) and are quantitatively the same as we found when the standard was composed of a common orientation (Fig. 3A and C, circles on far right).

**Fig. 4:**
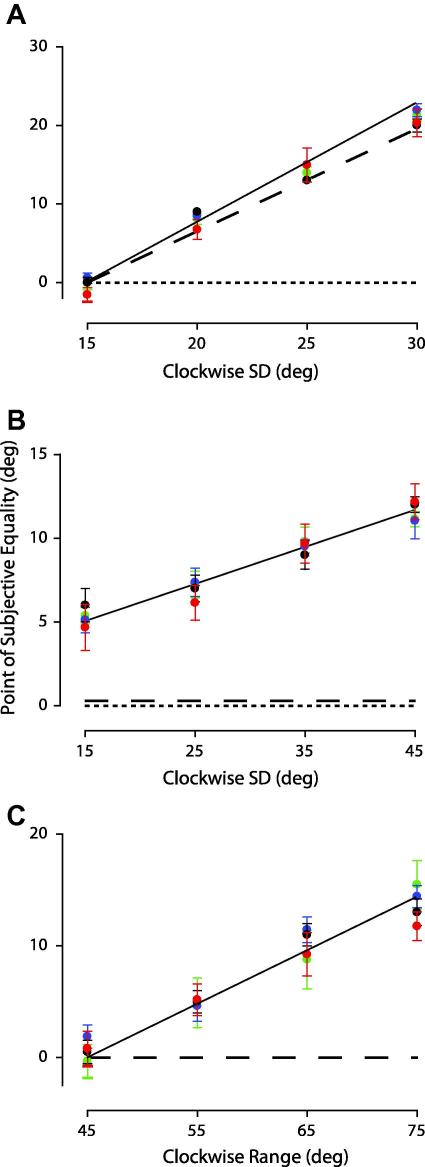
Relationship between perceived global orientation and *temporal* orientation statistics. Plotted and notated as in Fig. 3. The symbols and lines are the perceived and statistical orientations accumulated over time. Error bars: 95% CIs based on 5000 bootstraps.

We used exactly the same methods and analysis as described above in the temporal pooling experiments, with the exception that the texture patterns were dynamic and the orientations in the comparison were asymmetrically distributed over time rather than space. The perceived global orientation accumulated over time (point of subjective equality) is plotted in Fig. 4A–C as a function of the clockwise SD or range of the comparison texture. Although the data are slightly more variable, it is striking how similar the results are to the spatial case. The perceived global orientation corresponds very closely to the mean line orientation accumulated over time, diverging substantially from both the modal (Fig. 4A and B) and median temporal statistics (Fig. 4B and C).

**Fig. 5:**
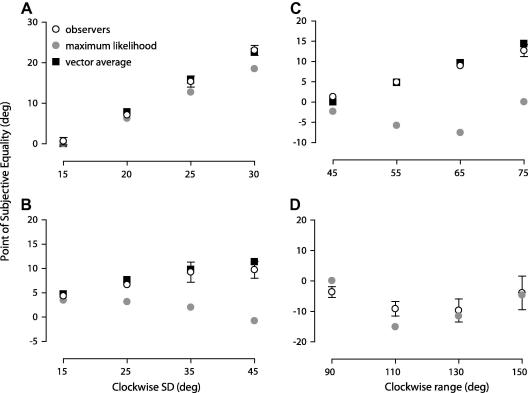
(A–C) Model simulations of the spatial pooling experiment. Plotted as in the same manner as Fig. 3. Vector average (black squares) and maximum likelihood (gray circles) estimate of the perceived global orientation of four observers (open circles; mean ± SD) in the spatial pooling experiment. (D) From Webb et al. (2007), perceived direction of motion of four observers (open circles; mean ± SD) plotted as a function of the ranges of the direction distributions used for the comparison stimulus. Gray circles show the global motion direction estimated by a maximum likelihood decoder.

We simulated observers’ performance on all of the experiments with a simple model, schematically illustrated in Fig. 1 and described in detail in Section 2. We read-off the perceived global orientation of texture patterns on each trial with a vector average, winner-takes-all and maximum likelihood decoder. The pattern of results we find in both the spatial and temporal experiments is accurately predicted by our simulations with a vector average, but not a maximum likelihood or winner-takes-all read-out of orientation signals. The results of the simulations for the spatial experiment are plotted in Fig. 5A–C. We only plot the estimates of the vector average (black squares) and maximum likelihood (gray circles) decoders, since winner-takes-all generated qualitatively similar predictions to maximum likelihood, but with higher variance. Open circles show the perceived global orientation in the spatial experiment for the four observers (mean ± SD) plotted and notated as in Figs. 3 and 4. The vector average response of the model neurons clearly provides a more accurate estimate of perceived global orientation than the other decoders. These results contrast with our previous work in which we found that both maximum likelihood and winner-takes-all provided a robust guide to the perceived direction of global motion (Webb et al., 2007).

In Fig. 5D, we plot the results of the experiment in our previous study which was most diagnostic for distinguishing the predictions of a vector average and maximum likelihood read-out of motion direction. The perceived direction of motion of four observers (open circles; mean ± SD) is plotted as a function of the ranges of the direction distributions used for the comparison stimulus. The filled circles show the global motion direction estimated by a maximum likelihood decoder, which clearly corresponds very closely to the perceived direction of the observers. It is notable how different these data are for the perceived direction of global dot motion (Fig. 5D) compared to that for global orientation (Fig. 5C), even though we used analogous stimulus distributions and psychophysical procedures in both studies.

**Fig. 6:**
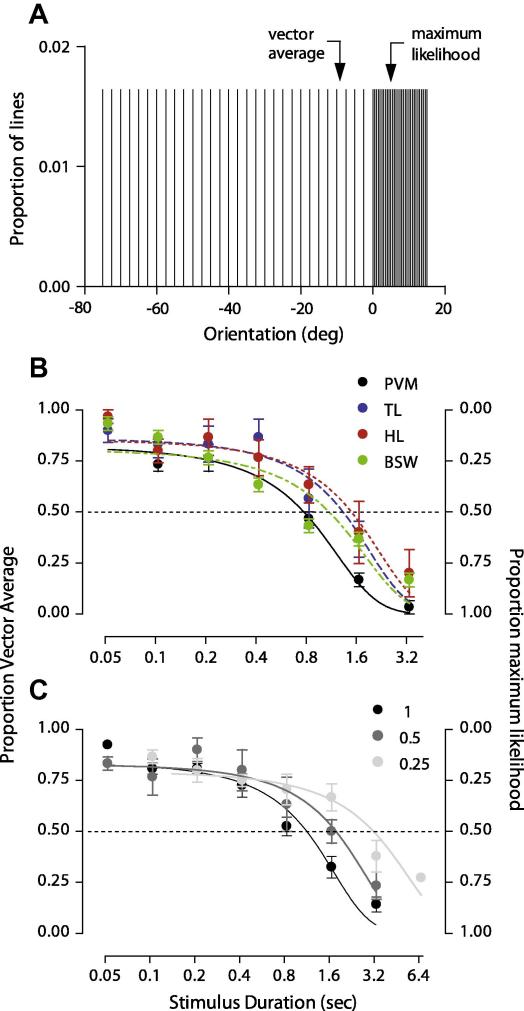
Biases in perceived global orientation as a function of stimulus duration and luminance contrast. (A) Skewed uniform distribution of orientations for which maximum likelihood and vector average decoders estimate the perceived global orientation to be clockwise and counter clockwise of vertical, respectively. (B) Colored symbols show for four observers the proportion of clockwise (maximum likelihood) and counter clockwise (vector average) judgements plotted as a function of stimulus duration. (C) Gray symbols show for four observers (mean ± SEM) how luminance contrast modulates the duration of the transition from a vector average to maximum likelihood read-out.

One important difference between the current and previous study, however, was that we used very different stimulus durations for the global motion and the orientation experiments. To try and reconcile these apparently conflicting results, we ran a simple experiment in which we varied stimulus duration. The observers’ task was to judge whether a static texture pattern composed of a skewed uniform distribution of orientations (shown in Fig. 6A) was oriented clockwise or counter clockwise of implicit vertical. We chose this distribution because in the model simulations maximum likelihood and vector average decoders estimated the perceived global orientation to be clockwise and counter clockwise of vertical, respectively. In Fig. 6B, we plot for four observers the proportion of clockwise (maximum likelihood) and counter clockwise (vector average) judgements as a function of stimulus duration. We have not presented the corresponding prediction of a winner-takes-all decoder because it is very similar to that of maximum likelihood. Perceived global orientation was biased towards a vector average read-out at the shortest stimulus durations tested and a maximum likelihood read-out at longer stimulus durations.

Similar biases away from a vector sum direction towards an IOC solution at certain durations have been found for type II plaid motion (Cropper, Badcock, & Hayes, 1994; Yo & Wilson, 1992). The duration-dependence of these effects was also modulated by stimulus contrast (Yo & Wilson, 1992). Here we test whether the duration of the transition between different read-outs of global orientation depends upon contrast. The task was the same as above: a clockwise or counter clockwise judgement about implicit vertical. Fig. 6C shows the average performance of four observers at three contrast levels. This plot shows that decreasing contrast increased the duration of the transition from a vector average to maximum likelihood read-out.

## 4 Discussion

We have examined which computations accurately predict the perceived global orientation of signals accumulated over space and time. Using a global orientation discrimination task, we found that the perceived global orientation of texture patterns coincided with the mean (or vector average read-out) of orientation signals. This result is consistent with a large body of work demonstrating that different forms of texture perception are well characterized by image-based, summary statistics (Balas, 2006; Balas et al., 2009; Beck, 1983; Dakin, 1999; Dakin & Watt, 1997; Julesz, 1981; Keeble et al., 1995; Kingdom et al., 2001; Morgan et al., 1998, 2008; Parkes et al., 2001; Voorhees & Poggio, 1988). We have, however, previously shown that spatial pooling of local samples might be better characterized in terms of optimal decoding of neuronal populations rather than image-based statistics (Webb et al., 2007). To reconcile the current results with our previous work, we varied the stimulus duration of texture patterns. Perceived global orientation was accurately predicted by a vector average read-out of orientation signals at relatively short stimulus durations and maximum likelihood read-out at longer durations. Moreover, decreasing the luminance contrast of texture patterns increased the duration at which the read-out translated from vector average to maximum likelihood.

The dynamics of the read-out of global orientation are reminiscent of earlier psychophysical work with certain types of two-dimensional motion (Cropper et al., 1994; Lorenceau, Shiffrar, Wells, & Castet, 1993; Yo & Wilson, 1992). At short stimulus durations (e.g. less than 90 ms) type II plaids are perceived moving in a vector average direction, whereas at longer durations they are perceived to move in a direction predicted by an IOC rule (Cropper et al., 1994; Yo & Wilson, 1992). The transition from vector average to an IOC direction of plaid motion over time is also modulated by luminance contrast (Yo & Wilson, 1992). Subsequent work demonstrated that many of these dynamic two-dimensional motion effects (Bowns, 1996; Burke & Wenderoth, 1993; Lorenceau et al., 1993; Stone & Thompson, 1992; Stone, Watson, & Mulligan, 1990; Yo & Wilson, 1992) can be explained within a Bayesian framework, provided one assumes that slow speeds are more likely (Weiss et al., 2002). We cannot rule out the possibility that an orientation equivalent of this framework with suitable, statistical *a priori* assumptions could predict our results.

One potential criticism of our interpretation is that the bias towards a maximum likelihood read-out at long stimulus durations can be explained by adaptation of neural responses in early visual cortex (Dragoi, Sharma, & Sur, 2000; Maffei, Fiorentini, & Bisti, 1973; Movshon & Lennie, 1979; Muller, Metha, Krauskopf, & Lennie, 1999). It is certainly true that the longer stimulus durations will have caused adaptation of the population response in early visual cortex. However, this would predict a relative reduction of the response to the denser part of the orientation distribution (shown in Fig. 6A), causing a bias away from a maximum likelihood read-out at long durations. Similarly, surround suppression of neural responses in early visual cortex (Blakemore & Tobin, 1972; Cavanaugh, Bair, & Movshon, 2002; DeAngelis, Freeman, & Ohzawa, 1994; Webb, Dhruv, Solomon, Tailby, & Lennie, 2005) cannot account for the bias at long durations, since this would also reduce responses most to the denser part of the distribution.

Analogous changes to the nature of the pooling computation have also been found to occur over different temporal scales in single neurons in the middle temporal (MT) area (Pack & Born, 2001; Smith, Majaj, & Movshon, 2005). In these studies, the responses of individual neurons were initially dominated by the component directions of a plaid pattern, and much later in the response by the pattern, or plaid direction of motion. These results closely mirror the psychophysics (Cropper et al., 1994; Yo & Wilson, 1992) and reinforce the notion that global stimulus selectivity takes time to evolve. At present, it is unknown whether or not the perceived direction of asymmetrical distributions of global motion direction is predicted by different forms of read-out at different stimulus durations. Based on the current results, we would predict that a vector average computation might be a better estimator of the perceived direction of global dot motion at very short stimulus durations. Ongoing work in our laboratory is testing this prediction (see below).

The concept of adaptive pooling – a flexible process in which the visual system recruits different estimators to address the prevailing computational demands – is gaining prominence in the literature (Amano, Edwards, Badcock, & Nishida, 2009; Bowns & Alais, 2006; Ferrera, 2000; Huang, Albright, & Stoner, 2007; Liu & Wang, 2008; Nichols & Newsome, 2002; Pack, Berezovskii, & Born, 2001; Pack & Born, 2001; Recanzone & Wurtz, 1999; Zohary et al., 1996). In a recent psychophysical demonstration of this phenomenon, Amano and colleagues found that the computations mediating spatial pooling of motion signals depend upon the available stimulus information. They showed that the visual system pools ambiguous local direction signals simultaneously across orientation and space in a manner consistent with IOC, whereas unambiguous motion is first pooled locally across orientation and then pooled globally across space according to a vector average computation. This reinforces the notion that pooling may not be a rigid process, but rather depends upon the nature of the stimulus and task demands.

Our current and previous results (Webb et al., 2007) suggest that spatial pooling of local direction and orientation use similar probabilistic read-out strategies when sufficient time is available. When time is limited, the visual system appears to adopt a parsimonious, but potentially biased read-out of visual signals. One possibility is that it generates its “best estimate” based upon the limited information available, but resorts to optimal pooling over longer time scales. For example, a vector average decoder might be deployed during the early phase of a neural response to rapidly initiate ocular motor systems (Ferrera, 2000; Groh et al., 1997; Huang & Lisberger, 2009; Lisberger & Ferrera, 1997; Masson, 2004; Osborne & Lisberger, 2009; Recanzone & Wurtz, 1999; Wallace et al., 2005; Yang & Lisberger, 2009) before complex stimulus selectivity has time to evolve (Cropper et al., 1994; Pack & Born, 2001; Smith et al., 2005; Yo & Wilson, 1992). Whereas, optimal, non-linear pooling can take time to evolve because of the dynamics of the underlying neural computations. Neural networks have to mitigate the effects of moment to moment fluctuations (i.e. neural noise) in the system, and it can take several iterations of activity before a recurrent network approaches an optimal decoding solution (Deneve et al., 1999).

An intriguing aspect of our results is the smooth transition from a vector average to maximum likelihood read-out over time. This gradual shift might reflect the implementation of two decoding operations that are weighted against each other. This sort of competitive inhibition implemented within a simple recurrent network can account for the gradual shift from a vector average to WTA read-out of ocular motor signals (Ferrera, 2000). An alternative proposal is that the transition might reflect a form of temporal summation. That is, each neuron in the population can only sum a fixed number of samples per unit time before its response reaches saturation – the orientation equivalent of a semi-saturation constant. Preliminary work in our laboratory suggests that implementing this form of temporal summation in our population decoding model can cause a maximum likelihood decoder to gradually change its read-out over time.

## References

[bib1] Adelson E.H., Movshon J.A. (1982). Phenomenal coherence of moving visual patterns. Nature.

[bib2] Alais D., Burr D. (2004). The ventriloquist effect results from near-optimal bimodal integration. Current Biology.

[bib3] Albright T.D. (1984). Direction and orientation selectivity of neurons in visual area MT of the macaque. Journal of Neurophysiology.

[bib4] Amano K., Edwards M., Badcock D.R., Nishida S. (2009). Adaptive pooling of visual motion signals by the human visual system revealed with a novel multi-element stimulus. Journal of Vision.

[bib5] Andersen J. (1989). Perception of three-dimensional structure from optic flow without locally smooth velocity. Journal of Experimental Psychology: Human Perception and Performance.

[bib6] Anstis S. (2009). ‘Zigzag motion’ goes in unexpected directions. Journal of Vision.

[bib7] Ariely D. (2001). Seeing sets: Representation by statistical properties. Psychological Science.

[bib8] Balas B.J. (2006). Texture synthesis and perception: Using computational models to study texture representations in the human visual system. Vision Research.

[bib9] Balas B., Nakano L., Rosenholtz R. (2009). A summary-statistic representation in peripheral vision explains visual crowding. Journal of Vision.

[bib10] Baldassi S., Burr D.C. (2004). “Pop-out” of targets modulated in luminance or colour: The effect of intrinsic and extrinsic uncertainty. Vision Research.

[bib11] Baldassi S., Megna N., Burr D.C. (2006). Visual clutter causes high-magnitude errors. PLoS Biology.

[bib12] Baldassi S., Verghese P. (2002). Comparing integration rules in visual search. Journal of Vision.

[bib13] Beck J. (1983). Textural segmentation, second-order statistics, and textural elements. Biological Cybernetics.

[bib14] Beck J.M., Ma W.J., Kiani R., Hanks T., Churchland A.K., Roitman J. (2008). Probabilistic population codes for Bayesian decision making. Neuron.

[bib15] Blakemore C., Tobin E.A. (1972). Lateral inhibition between orientation detectors in the cat’s visual cortex. Experimental Brain Research.

[bib16] Bowns L. (1996). Evidence for a feature tracking explanation of why type II plaids move in the vector sum direction at short durations. Vision Research.

[bib17] Bowns L., Alais D. (2006). Large shifts in perceived motion direction reveal multiple global motion solutions. Vision Research.

[bib18] Burke D., Wenderoth P. (1993). The effect of interactions between one-dimensional component gratings on two-dimensional motion perception. Vision Research.

[bib19] Cavanaugh J.R., Bair W., Movshon J.A. (2002). Selectivity and spatial distribution of signals from the receptive field surround in macaque V1 neurons. Journal of Neurophysiology.

[bib20] Chong S.C., Treisman A. (2003). Representation of statistical properties. Vision Research.

[bib21] Cohen E.H., Singh M., Maloney L.T. (2008). Perceptual segmentation and the perceived orientation of dot clusters: The role of robust statistics. Journal of Vision.

[bib22] Cropper S.J., Badcock D.R., Hayes A. (1994). On the role of second-order signals in the perceived direction of motion of type II plaid patterns. Vision Research.

[bib23] Dakin S.C. (1999). Orientation variance as a quantifier of structure in texture. Spatial Vision.

[bib24] Dakin S.C., Watt R.J. (1997). The computation of orientation statistics from visual texture. Vision Research.

[bib25] David S.V., Hayden B.Y., Gallant J.L. (2006). Spectral receptive field properties explain shape selectivity in area V4. Journal of Neurophysiology.

[bib26] DeAngelis G.C., Freeman R.D., Ohzawa I. (1994). Length and width tuning of neurons in the cat’s primary visual cortex. Journal of Neurophysiology.

[bib27] Deneve S., Latham P.E., Pouget A. (1999). Reading population codes: A neural implementation of ideal observers. Nature Neuroscience.

[bib28] Dragoi V., Sharma J., Sur M. (2000). Adaptation-induced plasticity of orientation tuning in adult visual cortex. Neuron.

[bib29] Edwards M., Greenwood J.A. (2005). The perception of motion transparency: A signal-to-noise limit. Vision Research.

[bib30] Ernst M.O., Banks M.S. (2002). Humans integrate visual and haptic information in a statistically optimal fashion. Nature.

[bib31] Fennema C., Thompson W. (1979). Velocity determination in scenes containing several moving objects. Computer Graphics and Image Processing.

[bib32] Ferrera V.P. (2000). Task-dependent modulation of the sensorimotor transformation for smooth pursuit eye movements. Journal of Neurophysiology.

[bib33] Foldiak P., Eeckman F., Bower J. (1993). The ‘ideal homunculus’: Statistical inference from neural population responses. Computation and neural systems.

[bib34] Georgopoulos A.P., Kettner R.E., Schwartz A.B. (1988). Primate motor cortex and free arm movements to visual targets in three-dimensional space. II. Coding of the direction of movement by a neuronal population. Journal of Neuroscience.

[bib35] Georgopoulos A.P., Schwartz A.B., Kettner R.E. (1986). Neuronal population coding of movement direction. Science.

[bib36] Gheri C., Baldassi S. (2008). Non-linear integration of crowded orientation signals. Vision Research.

[bib37] Gold J.I., Shadlen M.N. (2001). Neural computations that underlie decisions about sensory stimuli. Trends in Cognitive Sciences.

[bib38] Groh J.M., Born R.T., Newsome W.T. (1997). How is a sensory map read out? Effects of microstimulation in visual area MT on saccades and smooth pursuit eye movements. Journal of Neuroscience.

[bib39] Huang X., Albright T.D., Stoner G.R. (2007). Adaptive surround modulation in cortical area MT. Neuron.

[bib40] Huang X., Lisberger S.G. (2009). Noise correlations in cortical area MT and their potential impact on trial-by-trial variation in the direction and speed of smooth-pursuit eye movements. Journal of Neurophysiology.

[bib41] Jacobs R.A. (1999). Optimal integration of texture and motion cues to depth. Vision Research.

[bib42] Jazayeri M., Movshon J.A. (2006). Optimal representation of sensory information by neural populations. Nature Neuroscience.

[bib43] Julesz B. (1981). A theory of preattentive texture discrimination based on first-order statistics of textons. Biological Cybernetics.

[bib44] Keeble D.R., Kingdom F.A., Moulden B., Morgan M.J. (1995). Detection of orientationally multimodal textures. Vision Research.

[bib45] Kim J., Wilson H.R. (1993). Dependence of plaid motion coherence on component grating directions. Vision Research.

[bib46] Kingdom F.A., Hayes A., Field D.J. (2001). Sensitivity to contrast histogram differences in synthetic wavelet-textures. Vision Research.

[bib47] Knill D.C., Pouget A. (2004). The Bayesian brain: The role of uncertainty in neural coding and computation. Trends in Neurosciences.

[bib48] Landy M.S., Maloney L.T., Johnston E.B., Young M. (1995). Measurement and modeling of depth cue combination: In defense of weak fusion. Vision Research.

[bib49] Lisberger S.G., Ferrera V.P. (1997). Vector averaging for smooth pursuit eye movements initiated by two moving targets in monkeys. Journal of Neuroscience.

[bib50] Liu F., Wang X.J. (2008). A common cortical circuit mechanism for perceptual categorical discrimination and veridical judgment. PLoS Computational Biology.

[bib51] Lorenceau J., Shiffrar M., Wells N., Castet E. (1993). Different motion sensitive units are involved in recovering the direction of moving lines. Vision Research.

[bib52] Ma W.J., Beck J.M., Latham P.E., Pouget A. (2006). Bayesian inference with probabilistic population codes. Nature Neuroscience.

[bib53] Maffei L., Fiorentini A., Bisti S. (1973). Neural correlate of perceptual adaptation to gratings. Science.

[bib54] Masson G.S. (2004). From 1D to 2D via 3D: Dynamics of surface motion segmentation for ocular tracking in primates. Journal of Physiology – Paris.

[bib55] Mingolla E., Todd J.T., Norman J.F. (1992). The perception of globally coherent motion. Vision Research.

[bib56] Morgan M., Chubb C., Solomon J. (2008). A ‘dipper’ function for texture discrimination based on orientation variance. Journal of Vision.

[bib57] Morgan M., Ward R., Castet E. (1998). Visual search for a tilted target: Tests of spatial uncertainty models. Quarterly Journal of Experimental Psychology. A, Human Experimental Psychology.

[bib58] Motoyoshi I., Nishida S., Sharan L., Adelson E.H. (2007). Image statistics and the perception of surface qualities. Nature.

[bib59] Movshon J., Adelson E., Gizzi M., Newsome W., Chagas C., Gattass R., Gross C. (1986). The analysis of moving visual patterns. Pattern recognition mechanisms.

[bib60] Movshon J.A., Lennie P. (1979). Pattern selective adaptation in striate cortical neurones. Nature.

[bib61] Muller J.R., Metha A.B., Krauskopf J., Lennie P. (1999). Rapid adaptation in visual cortex to the structure of images. Science.

[bib62] Nichols M.J., Newsome W.T. (2002). Middle temporal visual area microstimulation influences veridical judgments of motion direction. Journal of Neuroscience.

[bib63] Osborne L.C., Lisberger S.G. (2009). Spatial and temporal integration of visual motion signals for smooth pursuit eye movements in monkeys. Journal of Neurophysiology.

[bib64] Pack C.C., Berezovskii V.K., Born R.T. (2001). Dynamic properties of neurons in cortical area MT in alert and anaesthetized macaque monkeys. Nature.

[bib65] Pack C.C., Born R.T. (2001). Temporal dynamics of a neural solution to the aperture problem in visual area MT of macaque brain. Nature.

[bib66] Palmer J. (1994). Set-size effects in visual search: The effect of attention is independent of the stimulus for simple tasks. Vision Research.

[bib67] Palmer J., Ames C.T., Lindsey D.T. (1993). Measuring the effect of attention on simple visual search. Journal of Experimental Psychology: Human Perception and Performance.

[bib68] Palmer J., Verghese P., Pavel M. (2000). The psychophysics of visual search. Vision Research.

[bib69] Paradiso M.A. (1988). A theory for the use of visual orientation information which exploits the columnar structure of striate cortex. Biological Cybernetics.

[bib70] Parkes L., Lund J., Angelucci A., Solomon J.A., Morgan M. (2001). Compulsory averaging of crowded orientation signals in human vision. Nature Neuroscience.

[bib71] Pavlovskaya M., Vol I., Blum B. (1992). Facilitation of pattern recognition by cuing foveation with the luminance centroid as origin of the frame of reference. Ophthalmic and Physiological Optics.

[bib72] Peirce J.W. (2007). Psychopy – Psychophysics software in Python. Journal of Neuroscience Methods.

[bib73] Pouget A., Dayan P., Zemel R. (2000). Information processing with population codes. Nature Reviews Neuroscience.

[bib74] Pouget A., Dayan P., Zemel R.S. (2003). Inference and computation with population codes. Annual Review of Neuroscience.

[bib75] Pouget A., Zhang K., Deneve S., Latham P.E. (1998). Statistically efficient estimation using population coding. Neural Computation.

[bib76] Recanzone G.H., Wurtz R.H. (1999). Shift in smooth pursuit initiation and MT and MST neuronal activity under different stimulus conditions. Journal of Neurophysiology.

[bib77] Regan D., Beverley K.I. (1985). Postadaptation orientation discrimination. Journal of the Optical Society of America A. Optics and Image Science.

[bib78] Salinas E., Abbott L.F. (1994). Vector reconstruction from firing rates. Journal of Computational Neuroscience.

[bib79] Salzman C.D., Newsome W.T. (1994). Neural mechanisms for forming a perceptual decision. Science.

[bib80] Sanger T.D. (1996). Probability density estimation for the interpretation of neural population codes. Journal of Neurophysiology.

[bib81] Seung H.S., Sompolinsky H. (1993). Simple models for reading neuronal population codes. Proceedings of the National Academy of Sciences of the United States of America.

[bib82] Shamir M. (2006). The scaling of winner-takes-all accuracy with population size. Neural Computation.

[bib83] Sharan L., Li Y., Motoyoshi I., Nishida S., Adelson E.H. (2008). Image statistics for surface reflectance perception. Journal of the Optical Society of America. A, Optics, Image Science, and Vision.

[bib84] Shaw M., Nickerson R. (1980). Identifying attentional and decision-making components in information processing. Attention and performance.

[bib85] Shaw M. (1982). Attending to multiple sources of information. I. The integration of information in decision making. Cognitive Psychology.

[bib86] Shiu L.P., Pashler H. (1995). Spatial attention and vernier acuity. Vision Research.

[bib87] Smith M.A., Majaj N.J., Movshon J.A. (2005). Dynamics of motion signaling by neurons in macaque area MT. Nature Neuroscience.

[bib88] Snippe H.P. (1996). Parameter extraction from population codes: A critical assessment. Neural Computation.

[bib89] Solomon J.A., Lavie N., Morgan M.J. (1997). Contrast discrimination function: Spatial cuing effects. Journal of the Optical Society of America. A, Optics, Image Science, and Vision.

[bib90] Stone L.S., Thompson P. (1992). Human speed perception is contrast dependent. Vision Research.

[bib91] Stone L.S., Watson A.B., Mulligan J.B. (1990). Effect of contrast on the perceived direction of a moving plaid. Vision Research.

[bib92] Treue S., Hol K., Rauber H.J. (2000). Seeing multiple directions of motion-physiology and psychophysics. Nature Neuroscience.

[bib93] Verghese P., Stone L.S. (1995). Combining speed information across space. Vision Research.

[bib94] Voorhees H., Poggio T. (1988). Computing texture boundaries from images. Nature.

[bib95] Wallace J.M., Stone L.S., Masson G.S. (2005). Object motion computation for the initiation of smooth pursuit eye movements in humans. Journal of Neurophysiology.

[bib96] Watamaniuk S., Duchon A. (1992). The human visual system averages speed information. Vision Research.

[bib97] Watt R.J., Morgan M.J., Ward R.M. (1983). Stimulus features that determine the visual location of a bright bar. Investigative Ophthalmology and Visual Science.

[bib98] Webb B.S., Dhruv N.T., Solomon S.G., Tailby C., Lennie P. (2005). Early and late mechanisms of surround suppression in striate cortex of macaque. Journal of Neuroscience.

[bib99] Webb B.S., Ledgeway T., McGraw P.V. (2007). Cortical pooling algorithms for judging global motion direction. PNAS.

[bib100] Weiss Y., Fleet D.J., Olshausen B., Lewicki M.S. (2002). Velocity likelihoods in biological and machine vision. Probabilistic models of the brain: Perception and neural function.

[bib101] Weiss Y., Simoncelli E.P., Adelson E.H. (2002). Motion illusions as optimal percepts. Nature Neuroscience.

[bib102] Whitaker D., McGraw P.V., Pacey I., Barrett B.T. (1996). Centroid analysis predicts visual localization of first- and second-order stimuli. Vision Research.

[bib103] Williams D., Sekuler R. (1984). Coherent global motion percepts from stochastic local motions. Vision Research.

[bib104] Wilson H.R., Ferrera V.P., Yo C. (1992). A psychophysically motivated model for two-dimensional motion perception. Visual Neuroscience.

[bib105] Wilson H.R., Kim J. (1994). Perceived motion in the vector sum direction. Vision Research.

[bib106] Yang J., Lisberger S.G. (2009). Relationship between adapted neural population responses in MT and motion adaptation in speed and direction of smooth-pursuit eye movements. Journal of Neurophysiology.

[bib107] Yo C., Wilson H.R. (1992). Perceived direction of moving two-dimensional patterns depends on duration, contrast and eccentricity. Vision Research.

[bib108] Zemel R.S., Dayan P., Pouget A. (1998). Probabilistic interpretation of population codes. Neural Computation.

[bib109] Zohary E., Scase M.O., Braddick O.J. (1996). Integration across directions in dynamic random dot displays: Vector summation or winner take all?. Vision Research.

